# Towards universal influenza vaccines?

**DOI:** 10.1098/rstb.2011.0102

**Published:** 2011-10-12

**Authors:** Ab Osterhaus, Ron Fouchier, Guus Rimmelzwaan

**Affiliations:** Department of Virology, Erasmus MC, Rotterdam, The Netherlands

**Keywords:** universal flu vaccines, influenza, seasonal flu vaccines

## Abstract

Vaccination is the most cost-effective way to reduce the considerable disease burden of seasonal influenza. Although seasonal influenza vaccines are effective, their performance in the elderly and immunocompromised individuals would benefit from improvement. Major problems related to the development and production of pandemic influenza vaccines are response time and production capacity as well as vaccine efficacy and safety. Several improvements can be envisaged. Vaccine production technologies based on embryonated chicken eggs may be replaced by cell culture techniques. Reverse genetics techniques can speed up the generation of seed viruses and new mathematical modelling methods improve vaccine strain selection. Better understanding of the correlates of immune-mediated protection may lead to new vaccine targets besides the viral haemagglutinin, like the neuraminidase and M2 proteins. In addition, the role of cell-mediated immunity could be better exploited. New adjuvants have recently been shown to increase the breadth and the duration of influenza vaccine-induced protection. Other studies have shown that influenza vaccines based on different viral vector systems may also induce broad protection. It is to be expected that these developments may lead to more universal influenza vaccines that elicit broader and longer protection, and can be produced more efficiently.

## 1. Introduction

Seasonal, avian and pandemic influenza are three manifestations of human influenza which all have a different aetiology. However, the differences between these forms of influenza are generally poorly understood and appreciated [1]. Seasonal influenza or ‘winter flu’ is an annually recurring acute respiratory disease caused by an influenza A or B virus that affects between 2.5 and 10 per cent of the population in the moderate climate zones every year in the winter months. Therefore, ‘winter flu’ is associated with a high burden of disease and especially people within the so-called high-risk groups are more likely to develop serious disease and complications leading to increased morbidity and mortality. Seasonal influenza viruses manage to persist in the human population by a process called ‘antigenic drift’, which is based on the high mutation rate that allows these viruses to escape continuously from the antibodies that are generated after each influenza virus infection. Avian influenza is a sporadic form of influenza caused by one of the many influenza A viruses from birds, which are transmitted to humans either directly or indirectly, e.g. through an intermediate mammalian host like the pig. Although avian influenza may be a serious disease in humans with a very high case fatality rate, which is largely dependent on the viral subtype involved, avian influenza viruses are not, or are not efficiently, transmitted from human to human. Therefore, the number of human cases and overall burden of disease worldwide are relatively limited. If, however, an avian influenza virus, through a process of re-assortment and/or continuous mutation, does acquire the ability to spread from human to human efficiently, it may cause a worldwide or pandemic outbreak of influenza. In the past century, more than 50 million people have died during the four influenza pandemics that hit the world in 1918 (‘Spanish flu’), 1957 (‘Asian flu’), 1968 (‘Hongkong flu’) and 2009 (‘swine or Mexican flu’), respectively. It is, however, estimated that between these pandemics at least the same number of people have died worldwide from seasonal influenza.

Although societal interventions, like the implementation of hygienic measures and the closure of schools and social events, may reduce the spread of influenza, medical interventions to combat disease largely rely on surveillance, rapid diagnostics, dedicated clinical care and the use of vaccines and antivirals. Preventive vaccination is by far the most cost-effective way to combat seasonal influenza, although there is also room for improvement. Vaccine effectiveness in preventing laboratory-confirmed influenza illness when the vaccine strains are well matched to circulating strains is 70–90% in randomized, placebo-controlled trials conducted among children and young healthy adults, but is lower among elderly or immunocompromised persons. In years with a suboptimal match, vaccine benefit is likely to be lower, although the vaccine can still provide substantial benefit, especially against more severe outcomes [2,3].

Combating sporadic human cases of avian influenza is largely dependent on veterinary control measures including the implementation of adequate surveillance and vaccination policies for poultry in endemic or at risk areas, and the implementation of poultry culling strategies [4–9]. Hygienic measures in endemic areas should further reduce the risk of human exposure. Rapid laboratory diagnosis and specialized clinical care with the use of the correct antiviral regimens are needed to treat infected patients and limit the case fatality rate [10]. Human vaccination against avian and pandemic influenza is much more difficult to realize since currently no registered human vaccines are available and the seasonal influenza vaccines offer no protection against viruses from other subtypes. Although the feasibility of pre-pandemic vaccination strategies against certain avian influenza viruses ‘with high pandemic potential’ has been explored, the unpredictability of the emergence of pandemic viruses seriously limits this approach. Therefore there is an urgent need for the development of new generation influenza vaccines that offer broader protection, not only to emerging drift variants of seasonal influenza viruses, but preferably also to influenza A virus of different influenza A virus subtypes that regularly infect birds and mammals and may be the basis of future influenza pandemics. More ‘universal vaccines’ that offer broader and long-lived protection should therefore be considered the major challenge for the development of new generation influenza vaccines.

## 2. Vaccination against seasonal influenza

Influenza vaccination is the most cost-effective way to reduce the disease burden from seasonal influenza. Inactivated seasonal influenza vaccines have been used for this purpose since the 1940s. They are administered intramuscularly and may be given to individuals from six months of age onward. Alternatively, live attenuated influenza vaccine (LAIV) and cold-adapted influenza vaccine are administered intranasally and have been used in some countries since the 1960s [3]. In many countries, special recommendations and guidelines are in place for the vaccination of individuals in the so-called high-risk groups for influenza. These individuals may suffer from more serious disease and are more prone to develop severe complications when infected with seasonal influenza viruses. These groups classically include patients with chronic cardiovascular disease, chronic airway disease, diabetes mellitus, chronic renal dysfunction, immunocompromised individuals and the elderly. As the worldwide burden of seasonal influenza disease is substantial, the World Health Organization (WHO) has indicated that member states should evaluate the cost-effectiveness of introducing influenza vaccination into national immunization programmes [3].

There are several more specific recommendations for vaccination coverage by the WHO and the European Commission which advocate ideally aiming at a vaccination coverage of 75 per cent in the high-risk groups. Although some countries in North America and Europe have now indeed achieved such coverage rates, in most countries seasonal influenza vaccination does not have such a high public health priority. In the USA and Canada, however, more universal vaccination strategies that target the whole population have now been adopted.

Inactivated and LAIV seasonal influenza vaccines that are used today are virtually all formulated with viral antigens produced in embryonated chicken eggs. This technology was developed more than 60 years ago and has so far not been replaced by state-of-the-art *in vitro* cell culture techniques. The virus that is produced in the allantoic fluid of the embryonated chicken eggs is subsequently purified and inactivated to serve as whole inactivated influenza vaccine, after detergent treatment as split whole virus influenza vaccine, or after additional purification as subunit influenza vaccines. Also, LAIVs are produced by propagating attenuated influenza viruses in embryonated chicken eggs. Vaccination is primarily aimed at the induction of protective antibodies directed against the haemagglutinin (HA) and neuraminidase (NA). Antibodies against the HA are generally considered to be the major correlate of protection against the disease (table 1).

**Table 1.** Arms of the adaptive immune system and their viral targets that contribute to protective immunity against influenza.

arm of the immune system	viral target	remarks	vaccine type
antibodies	HA	antibodies specific for the globular head containing the receptor binding region can neutralize the virus	any vaccine containing this component
		primary correlate of protection	
		vaccine-induced antibodies need to match the epidemic strain	
	NA	antibodies inhibit virus replication by inhibiting NA activity and spread of virus	any vaccine containing this component
		vaccine-induced antibodies need to match the epidemic strain	
	M2	relatively conserved basis for more universal vaccine? antibodies are not virus-neutralizing	specific immunogen targeting the induction of these antibodies
		protective effect involves antibody-dependent cell-mediated cytotoxicity	
	HA stem	relatively conserved basis for more universal vaccine?	specific immunogen targeting the induction of these antibodies
	NP	relatively conserved, protective effect demonstrated in the presence of virus-specific T cells	any vaccine containing this component
		mode of action largely unknown	
CD4^+^ T cells	all viral proteins	essential for mounting robust virus B cell and CTL responses	any vaccine containing or expressing viral proteins
		direct action against infected cells	
CD8^+^ T cells	predominantly internal proteins, e.g. NP and M1	internal proteins are relatively conserved therefore, majority of virus-specific CD8^+^ T cells are cross-reactive and contribute to heterosubtypic immunity	live attenuated vaccines vector vaccines expressing internal proteins DNA vaccines
		basis for more universal vaccine?	special adjuvant systems
		key role in elimination of virus- infected cells	
		efficient induction of CD8^+^ T cells requires endogenous antigen processing and presentation	

The main challenge in the production of seasonal influenza vaccines is to identify the influenza A and B viruses that will most probably be circulating in the next influenza season. A unique worldwide surveillance system coordinated by WHO that monitors and characterizes circulating seasonal influenza viruses in all continents serves to identify the most likely virus candidates that should be represented in the vaccine for the coming season. This happens twice per year for the Northern and the Southern Hemispheres, respectively. This system has recently been complemented with real-time monitoring and mathematical modelling systems for seasonal influenza virus strains as they emerge [11,12]. This system not only allows a more adequate identification, visualization and comparison of newly arising drift variants of seasonal influenza viruses, but also allows the actual measurement of antigenic distances between existing and newly emerging influenza viruses. These real-time data provide an increasingly important aid in the identification of the best-fitting viral vaccine strains for the next influenza season.

The safety record of seasonal influenza vaccines is exceptionally good. The most common adverse event associated with inactivated vaccines is local reactogenicity at the site of injection, while systemic reactogenicity such as fever or malaise is less common. Adverse events associated with LAIV are nasal congestion and symptoms of mild influenza such as headache, myalgias and fever. LAIV administration may cause increased risk of wheezing in young children and the elderly. For review, see [3].

Seasonal influenza vaccines have been shown to be effective, especially in young and healthy individuals. However, in immunocompromised individuals, most of whom are over-represented in the high-risk groups, the effectiveness of the currently used seasonal influenza vaccines is considerably lower. Therefore, improvement of the effectiveness of seasonal influenza vaccines for the high-risk groups is a major research priority. It may be anticipated that the efforts aimed at the improvement of the effectiveness and breadth of pre-pandemic and pandemic influenza vaccines, as discussed below, will also yield clues for the improvement of the effectiveness of seasonal influenza vaccines. For example, one of the seasonal influenza vaccines currently used in the elderly contains the adjuvant MF59 that is claimed to have an enhancing effect on the longevity of the antibody response in the elderly and may thus provide longer protection (G. Del Giudice 2010, personal communication). It should, however, be realized that adjuvants in general only provide limited immune enhancing effect in individuals who have already been primed by infection or vaccination [13–15]. Furthermore, the annually repeated use of an adjuvant in seasonal influenza vaccines might carry an additional accumulating risk of causing adverse events. For these reasons, the development of more broadly reactive and longer-acting seasonal influenza vaccines should also focus on additional and novel approaches that are also explored for the generation of new generation pre-pandemic and pandemic vaccines, as described in §3*a*.

Finally, it is important to highlight that animal models are crucial for the preclinical evaluation of novel candidate influenza vaccines. In most cases, a tier of multiple animal models is used before the evaluation of vaccine candidates in clinical trials is considered. Commonly, vaccines are tested for safety and efficacy in mice, ferrets and/or macaques [16].

## 3. Vaccination against pandemic influenza

### (a) Pre-pandemic and pandemic influenza vaccines

The main problem in developing and producing pre-pandemic or pandemic vaccines is that the virus that will cause the next pandemic is not known in advance. Given the unpredictable nature and high variability of influenza viruses, it is hard to anticipate which influenza virus from which animal species will be at its base. This was best illustrated by the pandemic preparedness efforts that were implemented by many, predominantly industrialized, countries in the past decade. In the prelude to the 2009 H1N1 influenza pandemic, the world largely focused on the pandemic threat posed by the highly pathogenic avian influenza virus of the H5N1 subtype (HPAI-H5N1 virus) which, since 2003, had claimed hundreds of victims from zoonotic transmissions, mainly in South East Asia, without being able to spread efficiently from human to human [17]. Pre-pandemic candidate H5N1 vaccines were developed and tested in clinical trials and pre-clinically in ferret vaccination challenge experiments (see below).

Even with the available technologies and the implemented pandemic preparedness plans, it took more than six months to produce the first vaccine doses against the unexpected 2009 pandemic H1N1 (pdm H1N1) virus. This virus was first detected in Mexico and emerged as the consequence of a reassortment event between Eurasian and North American swine viruses [18]. For those countries that were first hit by the emerging pandemic, like those in the Southern Hemisphere, but also for some countries in the Northern Hemisphere, the vaccines clearly came too late and well after the pandemic struck. For other countries, the 2009 pdm H1N1 vaccines became available just before or during the peak of the pandemic. It should, however, be emphasized that this pandemic outbreak of influenza was the first during which people, albeit only in certain countries, could actually be vaccinated with pandemic vaccines. If the pandemic virus had been identified one month earlier, this would have made a difference in terms of the number of people that could be vaccinated in time.

Besides having a surveillance and early warning system in place, the three key issues related to the eventual availability of pandemic influenza vaccines are (i) response time from the moment the pandemic virus is identified, (ii) vaccine production capacity, and (iii) efficacy as well as safety of the pandemic vaccines concerned. The last also requires special pandemic preparedness of regulatory authorities. With the currently available technologies, the response time until the first doses of vaccine become available is apparently more than six months, after which large-scale production of the vaccines will take many additional months. The overall worldwide production capacity for pandemic influenza vaccines is largely dependent on the production of seasonal influenza vaccines, which today may be estimated to approximate about 900 million doses of trivalent influenza vaccine per year. If all this capacity could indeed be used, about 2.7 billion doses of monovalent influenza vaccine could be produced. It has, however, become clear that with the currently used inactivated formulations of seasonal influenza vaccines, whole inactivated, split and subunit vaccines, which use about 15 µg of viral HA per vaccine dose, no protective immune response can be obtained in individuals who are naive towards the pandemic virus. Using avian H5N1 viral antigen in clinical trials, it was shown that two vaccinations with at least 90 µg of HA were needed to induce the required antibody response, although this dose could be considerably reduced by the inclusion of an adjuvant [19]. Obviously, this reduces the available pandemic influenza vaccine production capacity dramatically. Besides these key issues, there are numerous additional logistic problems in vaccinating whole populations against a rapidly spreading influenza virus. These should all be addressed in pandemic preparedness plans that, according to WHO recommendations, each country should have in operation.

## 4. Novel approaches using existing vaccine formulations

The influenza vaccine production technologies used today are virtually all based on the use of embryonated chicken eggs for the production of viral antigen. Obviously, this more than 60 year old approach has performed well over time but now clearly suffers from lack of flexibility and rapid up-scaling possibilities. Novel technologies that are now being explored and implemented, both in pre-clinical and clinical studies, include the use of continuous mammalian cell lines such as Madin Darby canine kidney (MDCK) cells, Vero cells and PERC-6 cells (table 2). Also, the use of influenza virus HA produced in plant cells or with baculovirus production systems are currently being tested. All these approaches may increase the availability of the production substrate.

**Table 2.** Recent developments to improve the timely availability of efficacious (pandemic) influenza vaccines.

	development	remarks
production of seed strains	use of reverse genetics	especially relevant for the preparation of safe vaccine strains against highly pathogenic avian influenza
		removal of basic cleavage site by site-directed mutagenesis
production platform	use of cell lines	various cell-culture systems have been established using MDCK, VERO or PERC6 cells
vaccine formulations	recombinant proteins	in addition to existing inactivated vaccine formulations (whole virus, split virion and subunit)
	live attenuated vaccines	production of recombinant proteins in insect cells using baculovirus expression system
		attenuation of live viruses by cold-adaptation (e.g. Flumist) or defective NS1 gene
increasing immunogenicity/dose sparing	use of adjuvants	use of oil-in-water adjuvants like MF59 and ASO3 improve immunogenicity
		allow dose sparing
alternative antigen delivery systems	establishment of viral vectors	recombinant adenovirus and poxviruses expressing influenza virus genes (e.g. HA)

Vaccine seed viruses are classically produced by reassortment methods using the circulating influenza viruses against which the vaccine should be produced together with production substrate-adapted influenza viruses. This is a time consuming and rather unpredictable procedure that is now being replaced by faster and more efficient reverse genetics techniques that have been developed in the past decade. The use of the novel reverse genetics approaches is subject to intellectual property rights.

Virus strain selection for seasonal influenza vaccines is based on the data generated by the influenza surveillance network that is coordinated by WHO through its worldwide network of affiliated national influenza centres and collaborating centres. On the basis of the epidemiological, virological and clinical data collected by the network, recommendations on vaccine strain selection are made twice per year to the vaccine manufacturers. In recent years, the surveillance system has been greatly improved by the implementation of real-time collection of the data and the use of antigenic cartography [20]. These methods are now also being implemented for the surveillance of avian and mammalian influenza viruses, which may be the basis of future pandemic viruses. Based on these data, repositories of animal seed strain viruses can be generated that serve to produce candidate pandemic vaccines rapidly when a pandemic virus arises from the animal world [21].

## 5. Novel approaches using newly identified correlates of protection

To date, the only correlate of immune-mediated protection against influenza that is used for the production and quality assessment of influenza vaccines is the induction of antibodies against the HA of the virus. It has, however, been demonstrated that the NA can also induce protective immunity (table 1). It was shown recently in ferret vaccination challenge experiments that candidate vaccines based on iscom-matrix-adjuvanted soluble HAs and NAs were equally protective against 2009 pdm H1N1 virus challenge. The inclusion of NA in a vaccine is therefore likely to reduce the HA dose required and to broaden the protective immunity [22].

To date, 16 HAs have been identified and only nine NAs (which are also more conserved than HAs), so the use of NA as an additional vaccine immunogen would probably be of interest. Although some regulatory authorities request the presence of NA in seasonal influenza vaccines, there are, unlike for the HA, no requirements for its quantification or quality assurance.

It was recently shown that a new generation of mouse and human virus neutralizing monoclonal antibodies, reactive with an epitope located in the stem region of the HA molecule and shared by the haemagglutinins of H1, H2, H5, H6 and H9 subtypes of influenza A virus, has prophylactic and therapeutic efficacy against severe HPAI-H5N1 virus infection in ferrets. This justifies the expectation that the epitope recognized by this antibody could also induce broad-neutralizing antibodies when incorporated as an immunogen in influenza vaccines aiming at a vaccine inducing broader cross-protection [23–25].

Another strategy that has been studied is targeting of the ectodomain of the M2 protein as an immunogen. M2 protein is a small membrane protein of influenza A viruses only, which upon natural infection does not induce protective antibodies. However, when used as an isolated immunogen, it has been shown to elicit protective antibodies in animal studies under certain conditions. This protection has been claimed to be broad as the M2 protein is relatively well conserved and therefore it could also contribute to the development of a more ‘universal vaccine’ [26].

Besides the induction of protective antibodies with the HA, the NA or the M2 proteins of influenza viruses, the induction of protective T cell responses should also be seriously considered (table 1). Although in recent years, it has been documented that protective T helper cell responses and cytotoxic T cell responses can be induced in animal model systems, the data on protective T cell responses in humans have remained more circumstantial for obvious reasons [27,28]. However, the identification of targets for the induction of protective T cell responses should also be considered an attractive approach. This would include the identification of internal viral proteins with conserved epitopes and the identification of efficient modes of delivery to induce these responses. These include the use of vector systems, live attenuated viruses, certain adjuvant systems and DNA-based vaccine candidates. As these approaches target predominantly conserved T cell epitopes, they may also be expected to contribute to the development of more universal or hetero-subtypic cross-protective vaccines. In several mouse models, it has been shown that pre-infection with a virus that shares all proteins with a lethal challenge virus but not the HA and the NA, may, in spite of not preventing considerable weight loss, still offer protection from a fatal outcome of the challenge infection [29]. Also when the internal viral proteins are only partially shared, protection from fatal challenge infection can still be observed [30]. The observed protection in these experiments has been shown to correlate strongly with virus-specific T cell responses including CD8^+^ T cell responses in, e.g. adoptive transfer and tetramer staining experiments.

In addition to these mouse data, it was shown in humans that cytotoxic T lymphocytes (CTLs) specific for, e.g. seasonal influenza viruses, display high cross-reactivity with HPAI-H5N1 viruses [31]. Collectively, these observations indicate that targeting cross-reactive CTL epitopes may also be a base for the development of a more universal influenza vaccine.

Although at least some of the T cell-mediated immunity induced by either vaccination or natural infection cannot be expected to be fully protective, it could still contribute to a significant degree of clinical protection, which for both seasonal and pandemic influenza vaccines may still be important.

## 6. Seasonal influenza vaccination and subsequent avian or pandemic influenza

In mouse and ferret experiments, it was recently shown that vaccination against seasonal H3N2 influenza virus protects against infection with this seasonal influenza virus but consequently also limits the induction of heterosubtypic T cell-mediated immunity that is elicited inefficiently by seasonal influenza vaccination. Animals vaccinated against seasonal H3N2 influenza virus prior to H3N2 influenza virus infection proved to be more susceptible to subsequent fatal infection with HPAI-H5N1 infection [32–34]. It has been speculated that a similar mechanism could underlie the observation that in some countries individuals vaccinated against seasonal influenza, but not against pandemic 2009 H1N1 influenza, appeared to be more susceptible to developing severe pandemic 2009 H1N1 influenza than those that had not been vaccinated against seasonal influenza [35–37]. Whether this was indeed the case is not clear at this moment, but it may still be concluded that children vaccinated against seasonal influenza should also be vaccinated against pandemic influenza when it arises. It should, however, be realized that with the currently available technology, a pandemic vaccine will not be available for some time after the pandemic starts. Therefore, the mechanism underlying this observation should be elucidated and in this light a thorough risk–benefit evaluation should be carried out when vaccination of all children against seasonal influenza, as is now recommended in some countries, is routinely implemented.

## 7. Novel approaches using adjuvants

Several adjuvants are used in animal vaccines with the aim of improving their protective efficacy in terms of level, breadth and duration of protection, at the cost of a relatively limited increase of adverse events. Although many veterinary vaccines used today are adjuvanted with more or less complex adjuvants or adjuvant systems, only a few seasonal influenza vaccines for humans are adjuvanted. This is in part related to the observation mentioned above, that adjuvants usually provide limited immune enhancing effect to vaccines in individuals who have already been primed by infection or vaccination. Although there are a large number of adjuvants and adjuvant systems that have shown promise in animal models, an unacceptable increase in adverse effects has in most cases limited their application in human vaccines. However, a new generation of proprietary oil in water adjuvants like MF59 and AS03 has shown great promise when tested pre-clinically in ferrets and clinically with HPAI-H5N1 antigens. After the start of the 2009 influenza pandemic, candidate vaccines formulated with these adjuvants were first tested in ferret challenge models. It was first shown that indeed two doses of as little as 2–4 µg of MF59- or AS03-adjuvanted pandemic vaccine would elicit complete protection from challenge with 2009 pdm H1N1 virus. In these experiments, it was also shown that vaccination with seasonal H1N1 vaccine was not protective, but did provide a significant priming effect to the pandemic vaccination in ferrets [38,39]. This was in agreement with the finding that relatively young people were more likely to develop serious 2009 pdm H1N1 virus infection than elderly people. It was subsequently shown in clinical studies that one vaccination with these adjuvanted vaccines was sufficient to reach the regulatory geometric mean titre (GMT), seroconversion rate (SCR) and seroprotection rate (SPR) thresholds. Close monitoring of the safety of the adjuvanted vaccines used during the last pandemic showed an overall expected and acceptable increase of local and systemic adverse events as judged by national and international evaluation bodies. An increased incidence of narcolepsy in children vaccinated with one of these adjuvanted vaccines in Finland and Sweden and possibly other European countries is currently under investigation [40,41]. About 250 cases of narcolepsy were identified among 20 million people who had been vaccinated with this vaccine, but a causal relationship has not yet been confirmed [42].

In conclusion, it may be stated that the new generation adjuvants have a clear antigen sparing effect on pandemic vaccines that is, however, less pronounced in individuals who have been primed earlier in their lives with antigenically related viruses or vaccines. It has, therefore, also led to a significant antigen sparing effect on 2009 pdm H1N1 vaccines.

## 8. Novel approaches using vectors for antigen delivery

Several replication-competent and -impaired viruses as well as bacteria and virus-like particles, plasmid DNA, autologous dendritic cells as professional antigen-presenting cells and exosomes have been studied as delivery vehicles for antigen delivery in animal models. Recombinant adenoviruses and poxviruses appear to be among the most promising candidates for both human and veterinary influenza vaccines. A promising candidate vaccine vector is modified vaccinia virus Ankara (MVA), originally developed as a safe smallpox vaccine, which can be exploited as a viral vector and has many favourable properties. It has been used to express influenza virus nucleoprotein, matrix protein and HA. These vaccine candidates have now been tested in several mouse, macaque and ferret vaccination challenge experiments in which it was shown that broad and long-lasting immunity against seasonal, HPAI-H5N1 and 2009 pdm H1N1 viruses could be induced with virtually no side effects [30,43–47].

Furthermore, the first clinical studies with a MVA candidate vaccine expressing the influenza virus nucleoprotein and the M1 protein have been performed and it was shown that this vaccine is immunogenic and elicited virus-specific CD8^+^ T cell responses [48].

## 9. Conclusion

Seasonal influenza vaccines have a good safety and effectiveness record. However, there is clearly room for improvement as these vaccines are least effective in those who need them the most: frail, elderly and immunocompromised individuals. In 2009, vaccines for pandemic influenza have for the first time in history become available during an influenza pandemic. Although these vaccines also proved to be highly effective and safe, for many countries they came too late or only became available well into the pandemic. For more than half a century, influenza vaccines have been produced with classical technology dependent on the use of embryonated chicken eggs, and the technologies used have so far have profited little from the revolution that is taking place in the field of vaccine biotechnology. However, adjuvanted influenza vaccines have been introduced for pandemic influenza and to a lesser extent for seasonal influenza, and several novel generations of influenza vaccines are currently being developed. This may eventually lead to broader and longer protective vaccines that can be produced faster and more efficiently. It may be expected that both seasonal and pandemic influenza vaccines will benefit greatly from these developments in the near future.
